# Detection and Characterization of Catechol Quinone-Derived Protein Adducts Using Biomolecular Mass Spectrometry

**DOI:** 10.3389/fchem.2019.00571

**Published:** 2019-08-21

**Authors:** Shu-Hui Chen, Chun-Wei Li

**Affiliations:** ^1^Department of Chemistry, National Cheng Kung University, Tainan, Taiwan; ^2^Department of Medical Imaging and Radiological Sciences, Kaohsiung Medical University, Kaohsiung, Taiwan

**Keywords:** catechol, quinone, catechol quinone, protein adduction, adductomics, biomolecular mass spectrometry, selective reaction monitoring, parallel reaction monitoring

## Abstract

The catechol quinone (CQ) motif is present in many biologically relevant molecules throughout endogenous metabolic products, foods, drugs, and environmental pollutants. The CQ derivatives may undergo Michael addition, and has been shown to yield covalent bonds with nucleophilic sites of cysteine, lysine, or histidine residue of proteins. The CQ-adducted proteins may exhibit cytotoxicity or biological functions different from their un-adducted forms. Identification, characterization, and quantification of relevant protein targets are essential but challenging goals. Mass spectrometry (MS) is well-suited for the analysis of proteins and protein modifications. Technical development of bottom-up proteomics has greatly advanced the field of biomolecular MS, including protein adductomics. This mini-review focuses on the use of biomolecular MS in (1) structural and functional characterization of CQ adduction on standards of proteins, (2) identification of endogenous adduction targets, and (3) quantification of adducted blood proteins as exposure index. The reactivity and outcome of CQ adduction are discussed with emphases on endogenous species, such as dopamine and catechol estrogens. Limitations and advancements in sample preparation, MS instrumentation, and software to facilitate protein adductomics are also discussed.

## Introduction

**Figure 1:**
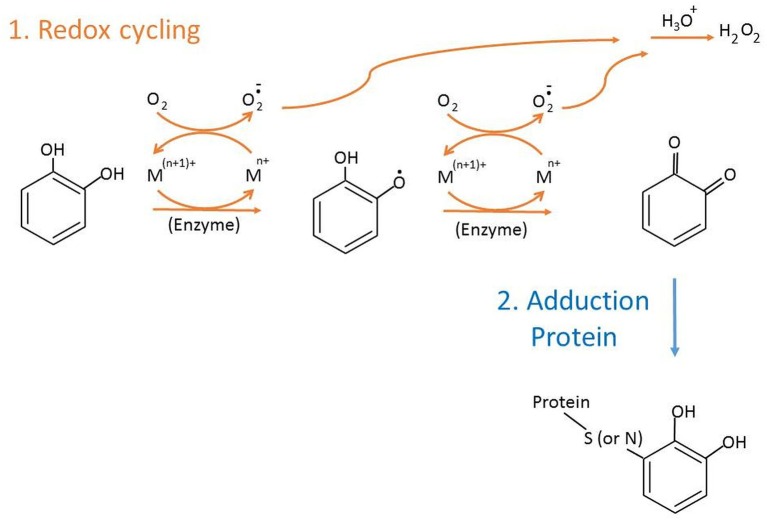
Two main reactions of catechol quinone.

Catechol-type polyphenols derived from endogenous metabolism or food/drugs are transformed to *o*-quinones accompanied by induced cellular bioactivities. In cells, derivatives of catechol quinones (CQs) undertake two major reactions (Figure 1). Firstly, one- or two-electron redox cycling (Liehr and Roy, 1990) generates reactive oxygen species, such as superoxides or hydrogen peroxide via autooxidation or by catalysis of enzymes or transition metals, like Cu^2+^ or Fe^3+^, which may change gene activation, bioreduction, or genotoxic effect. Another major reaction that competes with redox cycling of CQs is the covalent conjugation of *o*-quinone with nucleophilic sites on proteins or DNAs (Figure 1). If DNA adducts are not repaired or erroneously repaired, alterations in the DNA sequence may occur upon DNA replication, resulting in genotoxicity and cancer (Cavalieri et al., 2000, 2006). Compared to DNA, the biological effect of protein adduction by CQs is less understood. Detection and characterization of CQs-derived protein adducts are essential but challenging goals. In addition, the adduction level of major blood proteins such as hemoglobin (Hb) or human serum albumin (HSA) is a valuable marker of exposure level of the precursor molecules in the systemic circulation.

As previously reviewed (Törnqvist et al., 2002), protein adducts were mainly detected by isolation of modified amino acids or detachment of adducts from proteins followed by analysis with liquid chromatography (LC)-mass spectrometry (MS) or gas chromatography (GC)-MS analysis with or without derivatization. The emergence of proteomics has led to major technological advances in MS-based platform, greatly benefitting the detection and characterization of adducted proteins. The mass analyzers are gaining performance in resolution, speed, sensitivity, selectivity, robustness, and, equally important, user-friendliness for non-specialists (Ens and Standing, 2005; Zhang et al., 2013). Moreover, efficient and reliable search engines (Cox and Mann, 2008; Bruce et al., 2013; Lee et al., 2015), public data repositories (Perez-Riverol et al., 2015), and tools to characterize protein functions (Gavin et al., 2002) allow translation of MS data into meaningful biological information. These technical advancements have greatly enhanced the performance of non-targeted or targeted MS method and can significantly promote the impact of MS on protein adductomics. This review will focus on the biomolecular MS and proteomics approach for identification, characterization, and quantification of CQ-derived protein adducts.

## Reactivity of CQs TOWARD Proteins

It is known that *o*-quinones transformed from catechols are more electrophilic than their *p*-quinone isomers (Bolton and Shen, 1996; Iverson et al., 1996; Bolton et al., 1998) and may isomerize to give *p*-quinone methides, which are even more reactive electrophiles, in the presence of a suitable *para* substituent. Moreover, cyclic voltammetry measurements revealed that catechol estrogens with electron-donating substitutions are thermodynamically and kinetically more reactive than catechol molecules (Ku et al., 2016) and undergo autooxidation to form adducts under ambient conditions. The primary targets of CQ electrophiles are thiol and amine (lysine or histidine) functional groups. Disulfides were also identified to be targets of CQs (Fang et al., 2015). Kinetics data suggested that the reaction of 4-methyl-benzoquinone (4MBQ) with thiols was much faster than with amines (Li et al., 2016). The rate constant for the non-thiol protein α-lactalbumin was found to be 50- to 400-fold slower than that for benzenethiol or its derivatives at pH 7.0. Moreover, the reactivity with BSA decreased upon blocking Cys34 residue, the only free thiol on BSA (Li et al., 2016). Notably, unstable quercetin *o*-quinone derivative may reversibly isomerize to *p*-quinone methides, leading to interconversion between the two quercetin glutathione adducts formed (Boersma et al., 2000). Such thioether quinone conjugates were shown to exhibit addition–elimination reaction toward thiol groups of proteins (Li et al., 2005), indicating possibilities for release from the original adduction site and further electrophilic reactions of quinone methides at cellular sites different from those of its primary target (Boersma et al., 2000).

Although thiol groups are the main target sites of CQs, amines will become the main target for CQs when thiol groups are not available or blocked (i.e., oxidized or buried in the protein). Moreover, solvent accessibility, local pKa, microenvironment at the site of adduction, and the system generating the quinone or semiquinone species (Li et al., 2016) govern both the initial specificity and the structure of the final adduct. As confirmed by MS^2^ fragmentation, the N-terminal α-amino group reacted faster with 4MBQ than the side chain amine at pH 7.0, consistent with lower pKa for the α-carbon amine (Pierpoint, 1969). Moreover, the influence of pKa on reactivity is also exemplified by the increased reactivity for Lys residues in α-lactalbumin compared to the free amine, which may in part be explained by the lower pKa values (Gerken, 1984).

## Structural and Functional Characterization of CQ Adduction of Protein Standards

Many living systems generate CQ-containing species, including neurotransmitters (dopaquinone and dopamine quinine) and sex hormones (catechol estrogens). The ingredients of medicinal plants, herbs, and spices, such as tea catechin and flavonoid-derived or quercetin-containing CQ moieties are regarded as antioxidants that exhibit beneficial health effects (Jovanovic et al., 1994; Rice-Evans et al., 1996). However, protein adduction by CQs may cause adverse effects and exhibit different toxic end points in various species or cell systems (Macgregor and Jurd, 1978; Middleton, 1993). Table 1 summarizes examples of structural and functional characterization of CQ adduction of some protein standards using MS and other techniques. Major strengths of MS in these characterizations are intact molecular weight measurement for product detection and MS^2^ fragmentation for adduction site assignment. Functional changes induced by CQ adduction of these standards can be classified as (1) altered protein activity due to covalent conjugation with the free thiol, (2) altered protein stability due to modification of hydrophilic residues by lipophilic CQ derivatives, and (3) altered cell signaling due to hindered receptor or enzyme recognition domain. We have briefly discussed each of the functional changes.

**Table 1:** Structural and functional characterization of CQ adduction on protein standards involving MS.

**Protein standard (adduction site)**	**CQs**	**MS**	**References**
Glutathione (Cys)	Quercetin quinone methide, 4-methyl-1,2-benzoquinone, 4-methylcatechol	MALDI-MS LC-ESI-MS	Cheynier et al., 1986; Boersma et al., 2000
BSA	Chlorogenic acid, 4-methyl-1,2-benzoquinone, 4-methylcatechol	LC-ESI-MS	Rawel et al., 2002
α-Lactalbumin	Chlorogenic acid	MALDI-MS	Prigent et al., 2007
Lysozyme	Chlorogenic acid	MALDI-MS	Prigent et al., 2007
Myoglobin	o-Hydroxybenzene, gallic acid	MALDI-MS	Kroll and Rawel, 2001
GAPDH (Cys 152^*^)	Green tea polyphenol (–)-epigallocatechin-3-gallate (EGCG)	MALDI/TOF MALDI/TOFTOF	Ishii et al., 2008
Neuroglobin (Cys46, Cys55, and Cys120)	Catecholamines (dopamine, norepinephrine) and catechol estrogens (2-hydroxyestradiol and 4-hydroxyestradiol)	LC-ESI-MS^2^	Nicolis et al., 2013
Insulin (Cys 7A, Cys 7B, His 10B, Lys 29B)	Catechol estrogens (4-hydroxyestradiol)	LC-ESI-MS^2^	Ku et al., 2016
Cytochrome c (Cys 102, Lys7, Lys25, Lys39, Lys72, Lys87, and Lys88)	Benzoquinone, 2-(*N*-acetylcystein-*S*yl) benzoquinone, catechol estrogens	MALDI-MS LC-ESI-MS^2^	Guo et al., 2002; Fisher et al., 2007; Liang et al., 2018
Hemoglobin (N-termini, Lys62, α-Cys104, β-Cys93)	1,2- and 1,4-naphthoquinones, dopamine	LC-ESI-MS^2^ LC-MS^2^ including photodissociation	Zhang and Bartels, 2004; Diedrich and Julian, 2010

* *Identified by mutation*.

Free thiol is a reactive functional group and plays critical roles in enzyme activity and detoxification. The CQ–thiol conjugation affects the activity of thiol enzymes by forming covalent bonds with its cofactor-binding site. Glyceraldehyde-3-phosphate dehydrogenase (GAPDH), a thiol enzyme involved in catalyzing glycolysis, was identified as a target of polyphenol (–)-epigallocatechin-3-gallate (EGCG) in cancer cells (Ishii et al., 2008). Moreover, matrix-assisted laser desorption ionization–tandem time of flight (MALDI-TOF/TOF) detection and mutation experiments revealed that green tea EGCG is primarily conjugated with the cysteinyl thiol group of the active center of GAPDH, leading to irreversible inhibition of GAPDH activity (Ishii et al., 2008). Similarly, high-performance liquid chromatography (HPLC) coupled with MALDI-TOF and LC-MS^2^ detected that glutathione (GSH) conjugated with quercetin *o*-quinone through its free thiol (Boersma et al., 2000). Interestingly, the binding of EGCG with GAPDH could not be completely inhibited by the presence of a lower (1 mM) concentration of GSH (Ishii et al., 2008), implying that rapid CQ–thiol reactions have the potential to affect the high concentration of the reduced thiol pool (5 mM GSH in cells) and deplete the detoxification capability of cells. Neuroglobin (Ngb) is also thought to play a neuroprotective role as a scavenger of toxic reactive species. Using LC-MS^2^ analysis of the digested product, Cys120 of Ngb was identified to conjugate with catecholamine (Nicolis et al., 2013). However, catecholamines can form catechol oligomers, possibly through disubstituted quinol amine linkages. Ngb modifications by the monomeric catechol compounds, identified by tandem MS analysis, are likely to represent the initial stage of a multistep pathway leading to modifications by catechol oligomers and irreversible deterioration observed in Parkinsonian brains over long periods (Nicolis et al., 2013).

Unlike hydrophilic catecholamine, protein modification mediated by bulky and lipophilic catechol estrogen was less pronounced in forming oligomers, but strongly compromised protein stability through alteration of its interactions with solvent (Nicolis et al., 2013). Using LC-MS^2^ analysis of the digested product, Cys120, Cys46, and Cys55 of the reduced human Ngb were identified to conjugate with catechol estrogens. In spite of the lack of modification with oligomers, the solvent exposure of the Ngb-catechol estrogen, inferred from unfolding experiments, changed dramatically compared to Ngb-catecholamine, indicating that bulkiness and lipophilicity of catechol estrogen moieties have a great impact on the protein structure and stability.

The CQ adduction could sterically block or hinder the recognition domain for receptor binding or complex formation, resulting in altered cell signaling. Although insulin does not contain free thiols, adduction on disulfide linkage between Cys7 of the A and B chain, as well as at His10 or Lys29 in the B chain by catechol estrogens, was identified by LC-MS^2^. Moreover, CQ adduction on these sites was shown to lower insulin signaling and glucose uptake in MCF-7 cells (Ku et al., 2016), indicating reduced receptor recognition. Cytochrome c (cyt c), which is an electron carrier, was identified to be a target of catechol estrogens in rat liver microsome by proteomics (see discussion below). However, the addition site appeared to vary with different isoforms. In yeast cyt c isoform-1, Cys102 was reported to be modified by 1,4-benzoquinone using LC-MS^2^ (Louie and Brayer, 1990). Benzoquinone and 2-(*N*-acetylcystein-*S*yl) benzoquinone were reported to preferentially bind to solvent-exposed lysine-rich regions of horse heart cyt c, including Lys7, Lys25, Lys39, Lys72, Lys87, and Lys88 (Fisher et al., 2007), among which K7, K25, K39, and K72 (Yu et al., 2001) were identified to affect the Apaf-1 binding to cyt c. Such binding is an important initiating event in the mitochondria-controlled apoptotic pathway. The adducted cyt c was shown to be unable to initiate caspase-3 activation in native lysates and also inhibited Apaf-1 oligomerization into an apoptosome complex in a purely reconstituted system (Fisher et al., 2007).

## Identification of Protein Targets of CQs by Proteomics

Elucidation of the mechanism of action of active electrophiles remains an important area that requires further research. Searching for high-affinity proteins that conjugate to CQs is the first step in understanding the mechanism and impact of CQ adduction in cells. Proteomics techniques using LC-MS^2^ coupled with enzyme digestion and bioinformatics tools (bottom-up proteomics) offer a unique platform for the discovery of various protein modifications. The use of scoring algorithms for pattern recognition of MS^2^ spectra provides specificity and high confidence for identifying endogenously adducted proteins (Hansen et al., 2001).

Bottom-up proteomics was applied to identify endogenous (*in vivo*) and site-specific protein adduction by catechol estrogen in human blood (Fang et al., 2015). From an insulin resistance patient's blood, endogenous adduction of HSA and IgG1 at multiple Cys or Lys sites was initially identified by database search and confirmed by retention time and spectra matching with adducted protein standards. Using Ellman's test, catechol estrogen adduction was shown to produce stable products and irreversibly abolish the reactivity of Cys34 of HSA (Fang et al., 2015). So far, this is the only report on unambiguous identification of endogenous CQ adduction in blood proteins.

It is still challenging to identify endogenously adducted proteins because they are much less abundant than their unmodified forms, and often present below the detection limit of LC-MS^2^. Instead of endogenously modified proteins, chemical proteomics using electrophilic probe coupled with affinity chemistries, bottom-up proteomics, and software has been applied to explore potential protein targets of CQs in cells. A biotinylated 3,4-dihydroxyphenyl acetic acid (Bio-DPA) was used as a general probe to identify target proteins modified by catechol-type polyphenols in RL34 cells, and β-actin and Keap1 were identified to be the plausible targets of CQs (Ishii et al., 2009). Ethynyl estradiol (EE2), an endogenous estrogen, was used as the precursor probe to capture protein targets of catechol estrogens via *in situ* metabolic conversion and click reaction in rat liver microsomes (Liang et al., 2018). In order to differentiate the binding strength of captured targets, quantitative proteomics using stable isotope dimethyl labeling (Hsu et al., 2003) was applied. A total of 334 liver proteins were repeatedly identified and quantified, among which 259 proteins were classified to be covalent binders. Many identified proteins with covalent binding of adducts, including cyt c and many enzymes, are involved in cellular redox processes or detoxification activities. Moreover, such adduction was shown to suppress the superoxide oxidase activity of cyt c (Liang et al., 2018). Standard MS-based quantitative proteomics with 2-D gel electrophoresis or stable isotope labeling, which is not covered in this review, has been used as a platform to identify differential protein expression caused by CQs, like dopamine (Van Laar et al., 2008).

## Quantification of Adducted Proteins as Exposure Index

Owing to relatively high stability and long lifetime, quantifying adducted blood proteins (mainly HSA and Hb) is an excellent approach to monitor cumulative exposure to carcinogens or active electrophiles. Based on selective reaction monitoring (SRM) detection of the detached small molecules, estrogen quinone-derived protein adducts were previously shown to be biomarkers of estrogen homeostasis and a risk factor for developing breast cancer (Waidyanatha et al., 2002; Wang et al., 2006; Chen et al., 2011; Lin et al., 2014, 2018). However, this method lost the identity of adduction sites, which are important for the structural characterization and identification of new markers. Untargeted analytical schemes and bioinformatics pipeline coupled with the SRM or parallel reaction monitoring (PRM) method have been attempted for detecting and annotating various HSA–Cys34 adducts (Li et al., 2011; Grigoryan et al., 2016).

Cys34 is the only thiol group of HSA, and thus, the most likely adduction site of HSA. After tryptic digestion, HSA–Cys34 adducts are contained in the third largest tryptic peptide (T3) containing 21 amino acids with an average mass of 2433.87 Da. A fixed-step SRM method, which used a list of theoretical T3-adduct *m/z* values at a fixed increment between 9.1 and 351.1 Da of added masses to detect un-targeted adducts, was reported (Li et al., 2011). The method was shown to successfully detect standard adducts in a test mixture composed of various synthetic T3–Cys34 adducts, including estrogen quinone-T3, diol of estrogen quinone-T3, and oxidized estrogen quinone-T3. About 66 putative T3–Cys34 adducts out of a possible 77 candidates, covering a wide range of concentrations, were detected from six specimens pooled by gender, race, and smoking status. Most abundant adducts were found in the mass range below 100 Da, and they were more abundant in the circulating blood of smokers than in non-smokers (Li et al., 2011). Another pipeline reported by the same group combined accurate mass, tandem mass spectra, retention time, elemental composition, and database searches to characterize 43 Cys34 adducts in HSA in plasma from healthy individuals and several were found to be highly correlated with smoking status, race, and other variables (Grigoryan et al., 2016).

## Technical Limitations and Future Advancements of Adductomics for CQ Adducts

Identification, characterization, and quantification of adducted proteins from protein standards or complicated biological specimens by biomolecular MS still present significant challenges. Firstly, unlike enzyme-engaged post-translational modifications, electrophile-adducted modifications may not have a conserved sequence for the modification site and occur at multiple residues. This makes the search algorithm more complicated as a big database is generated if all possible variables are included. Alternatively, a multi-step search algorithm may be applied to increase the identification rate (Liang et al., 2018). Secondly, like many redox electrophiles, CQ conjugation is likely to suffer from artifacts during sample preparation. To minimize oxidation caused by Fenton chemistry, methanol was used as the primary solvent, and EDTA was added to the digestion buffer to capture metal ions, which may catalyze oxidations (Mir et al., 2014). Moreover, artifacts caused by metal adducts (Wilm and Mann, 1996; Karas et al., 2000) were reduced by replacing electrospray ionization (ESI) with nanoESI. In some studies, adduct enrichment, buffer exchange, and peptide fractionation steps were eliminated completely, and digestion was performed in the absence of reducing agents (Grigoryan et al., 2016).

Finally, like many post-translational modifications, the bottom-up approach will lose some adducted peptides due to incomplete digestion arising from hindered enzyme recognition domain or incomplete unfolding arising from insensitivity to a hydrophilic denaturant, like guanidinium. Such influences will become more pronounced when oligomers are formed like catecholamine, leading to the identification of fewer conjugation sites (Nicolis et al., 2013). The top-down approach is less likely to lose adducted species. However, precise adduction site identification is more difficult with the top-down approach. Photo-excitation of the quinone-adducted species appeared to be superior to CID in selectivity and simplicity, and the value becomes more apparent in whole proteins (Diedrich and Julian, 2010). Cleavage of the protein backbone of dopamine-modified alpha hemoglobin was observed selectively at a single Cys out of 140 residues of the whole protein. Moreover, a quinone-specific parent ion loss accompanied by additional d-fragments allowed a dopamine-modified Cys site to be assigned at the protein level (Diedrich and Julian, 2010).

Since the adduction level for CQs is expected to be below parts-per-million level, detection sensitivity is one of the most important bottlenecks for not only the top-down but also the bottom-up approach. Enrichment of adducted species for high recovery yield, without causing artifacts, remains to be important. Based on adduction levels detected from plasma specimens stored for 13 years at −80°C, artifact formation arising from collecting, storing, and processing biospecimens was claimed not to obscure meaningful comparisons in adductomic investigations (Grigoryan et al., 2016), implying that the stored biospecimens may always be re-examined using more sensitive techniques.

## Conclusions

The CQ adduction constitutes a risk of cytotoxic and genotoxic effects of cells. Biomolecular MS and proteomics techniques, which can be widely adopted to explore adductomics of different electrophiles or in different biological systems, can open a new era for adductomics by revealing CQ–protein interactions on a large scale and in great detail. Although technical innovations to improve precise identification, characterization, and quantification with low detection limits are still highly desirable, promising data summarized in this review warrant further explorations in CQ adduction using biomolecular MS.

## Author Contributions

C-WL was responsible for literature search, figure, table construction, and proof reading. S-HC was in charge of writing and submission.

### Conflict of Interest Statement

The authors declare that the research was conducted in the absence of any commercial or financial relationships that could be construed as a potential conflict of interest.
